# Selective Androgen Receptor Modulator Induced Hepatotoxicity

**DOI:** 10.7759/cureus.22239

**Published:** 2022-02-15

**Authors:** Sohaib Khan, Jaclyn Fackler, Asma Gilani, Stephanie Murphy, Lirio Polintan

**Affiliations:** 1 Internal Medicine, Parkview Medical Center, Pueblo, USA; 2 Gastroenterology, Parkview Medical Center, Pueblo, USA

**Keywords:** hepatotoxicity, acute hepatotoxicity, anabolic androgenic steroid, us fda, idiosyncratic drug reaction, androgen receptor, selective androgen receptor modulator

## Abstract

Selective androgen receptor modulators (SARMs) have been developed as an alternate to traditional anabolic steroids due to their favorable effects on the bones and muscles without androgenic side effects. They are very popular among athletes and bodybuilders and are available online or over the counter. The FDA has warned of their side effects including liver injury. Here we present the case of a 29-year-old patient who presented with jaundice, fatigue, and elevated liver function tests after starting SARM supplements. His symptoms improved and eventually resolved with stopping the supplements. The purpose of this case report is to raise awareness and educate clinicians of the potential side effect of hepatotoxicity of these supplements that can help in its early identification and management.

## Introduction

There are numerous anabolic substances and muscle growth promoters that have been developed for the management of muscles, bones, and disorders causing functional decline [1]. The anabolic substances include anabolic androgenic steroids (AASs) such as testosterone, growth hormone, and selective androgen receptor modulators (SARMs) [1]. These substances act on androgen receptor (AR), which are part of a superfamily of steroid receptors and have important effects on bone density, muscle mass, coagulation, strength, cognition, and male sexual development. AASs have numerous clinical applications. Their effects on the body can be broadly categorized as androgenic (impaired fertility, acne, virilization, prostate enlargement) and anabolic (effect on muscle mass, bone density, etc.) [2]. The negative androgenic effects have reduced their clinical application [3] and subsequently contributed to the formation of SARMs, which have a nonsteroidal structure [1]. SARMs are small molecule drugs that bind as a ligand to the ARs, causing tissue selective activities, which include both antagonist and agonist effects on ARs [2,4]. Unlike AASs, which have both anabolic and androgenic properties, SARMs express positive effects on muscles and bones without causing any androgenic effects of AASs. Due to these favorable properties, they are also marketed by some supplement manufacturers as legal steroids and also being desiccated for the management of osteoporosis, cachexia, and sarcopenia [4-6]. Although FDA and world anti-doping agency have not approved the use of SARMs, they are very popular among athletes and are heavily promoted as AAS alternatives [3,5]. Like AASs, which have hepatotoxic potential, there is only limited safety data available for SARMs. In 2017, due to extensive use of SARMs as nutritional supplements, FDA issued a warning about their serious health risks including liver toxicity [1,3,5]. Here we report a case of SARM supplement induced liver injury in a young patient.

## Case presentation

A 29-year-old male patient, bodybuilder, with no significant past medical history, presented to the hospital due to concerns of painless jaundice, pruritus, fatigue, and labs demonstrating significantly elevated liver function tests (LFTs). The patient reported that he noticed jaundice approximately five to six days prior to presentation, which was associated with scleral icterus, light-colored stools, dark urine, and significant fatigue. He otherwise was feeling well and denied any abdominal pain, nausea, vomiting, diarrhea, constipation, or signs of bleeding. There was no history of recent travel, new restaurants or food from a new source, IV drug abuse, blood transfusion, or getting a tattoo nonprofessionally. He reported no known family history of liver or autoimmune diseases or early onset chronic obstructive pulmonary disease. He used to drink a beer twice weekly but denied smoking or using any illicit drugs. He denied any recent medication change, but upon further investigation, it was found that patient started taking a SARM supplement approximately four weeks prior for bodybuilding. He also reported taking some pre-workout drinks.

Upon presentation, his blood pressure was 148/108 mm Hg, pulse was 77 beats/minutes, respiratory rate was 18/minutes, saturating was 98% on room air, and a temperature of 97.8°F. On examination, the patient was alert, oriented to time, place, and person, and without any acute distress. Scleral icterus and jaundice were visible on eye and skin examination. Cardiac examination showed a regular rate and rhythm without any murmurs, rubs, or gallops. Lung examination showed clear normal breath sounds without any rales, wheezing, or rhonchi. Abdominal examination demonstrated a soft, non-tender, non-distended abdomen with normal bowel sounds without any visceromegaly.

**Figure 1:**
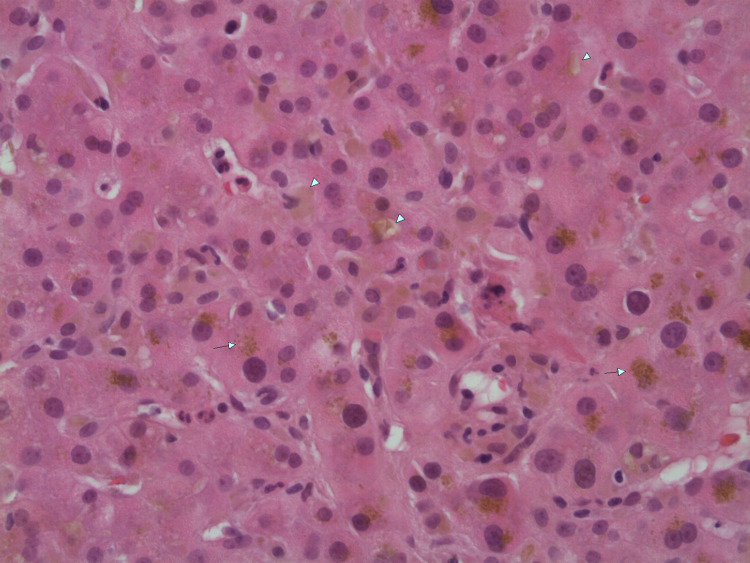
H&E stain at 400x magnification showing bile stasis (arrowheads) and lipofuscin pigment (arrows). H&E, hematoxylin and eosin

**Figure 2:**
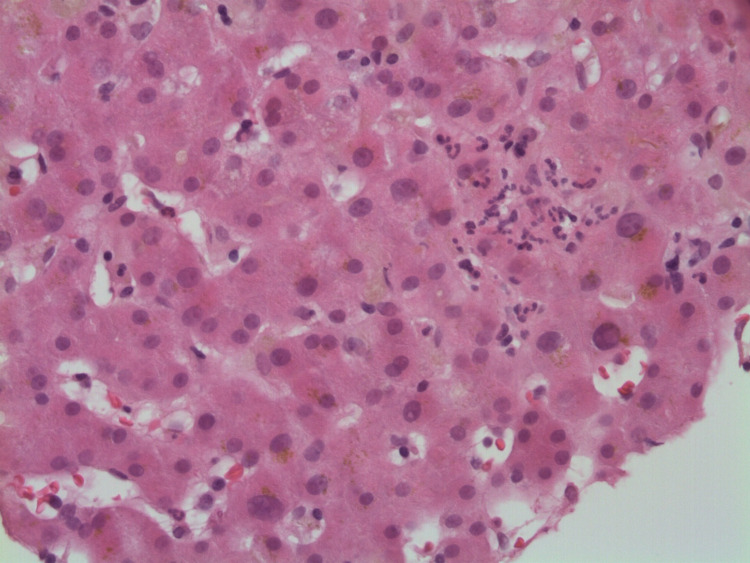
A collection of neutrophils (microabscess) present within the hepatic lobular parenchyma. Bile stasis and lipofuscin pigment are also present.

**Figure 3:**
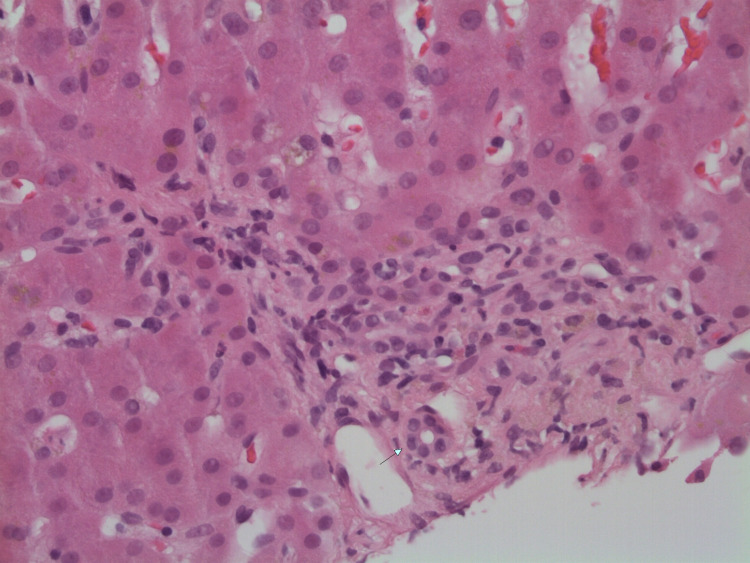
Portal tract showing sparse inflammatory cells and some cholestatic pigment within macrophages. Arrow denotes intact bile duct.

Initial laboratory studies were significant for hematocrit of 51.9%, platelet count of 470/μL, total bilirubin of 16.9 mg/dL, direct bilirubin of 12.1 mg/dL, AST (aspartate transaminase) of 79 U/L, ALT (alanine transaminase) of 165 U/L, and ALP (alkaline phosphatase) of 213 U/L. An iron panel showed iron of 176 mcg/dL, TIBC (total iron-binding capacity) of 428 mcg/dL, iron saturation of 41%, and ferritin of 602.1 ng/mL. Immunology, serology, and toxicology panels were normal without any significant findings. An abdominal ultrasound showed some intrahepatic biliary dilation without any extrahepatic dilation. A subsequent three-phase CT scan of the abdomen showed no evidence of hepatic/pancreatic mass, intra/extrahepatic biliary dilation, or abdominal lymphadenopathy. The patient then underwent biopsy of the right hepatic lobe (Figures 1-3), which showed centrilobular bile stasis with lipofuscin pigment along with collection of neutrophils (microabscesses) within lobular parenchyma. There was no significant portal, interface, or parenchymal inflammatory component, and portal tracts showed only sparse inflammatory cells. The portal tracts displayed a normal complement of bile ducts with no significant periductal onion skin fibrosis or granulomatous inflammation. Centrilobular intracytoplasmic bile pigment was noted. The sinusoids and central veins displayed no significant dilatation. An iron stain was negative for iron pigment. PAS staining with and without diastase was appropriate. A trichrome stain highlighted no significant portal, pericellular, or bridging fibrosis.

The patient was managed on supportive care with avoidance of any hepatotoxins. With normal imaging, serology, immunology, and a cholestatic pattern on liver biopsy, a diagnosis of drug-induced liver injury (DILI) was made secondary to the use of SARM supplements. The patient was discharged home with recommendations to stop taking the drug, monitoring of LFTs, and following up at the outpatient GI department.

At one week follow-up, the patient reported that he stopped taking SARM supplements and is also avoiding hepatotoxic medications such as Tylenol. He denied any nausea, vomiting, or abdominal pain, but he continued to have jaundice, easy fatigability, and pruritus, which responded to Benadryl. LFTs during this visit demonstrated total bilirubin of 22.6 mg/dL, ALP of 305 U/L, AST of 64 U/L, and ALT 119 of U/L. At four weeks follow-up, he reported improvement in his symptoms with LFTs showing total bilirubin of 3.5 mg/dL, ALP of 205 U/L, AST of 135 U/L, and ALT of 173 U/L. At two months follow-up, hepatic function panel improved with total bilirubin of 0.8 mg/dL, ALP of 138 U/L, AST of 37 U/L, and ALT of 70 U/L, and the patient reported resolution of jaundice, pruritus, and fatigue. At six months, LFTs normalized with total bilirubin of 1.1 mg/dL, ALP of 90 U/L, AST of 23 U/L, and ALT of 19 U/L. The patient is currently doing well and is avoiding any type of bodybuilding supplements.

## Discussion

SARMs are non-steroidal, small molecule, tissue selective androgen derivatives and can apply varying degree of both agonist and antagonist effects on AR in different tissues [2,5,7]. They perform as a ligand that moves into the cell by diffusion and binds to the AR in the cytoplasm forming receptor-ligand complex. This receptor-ligand complex then translocates to the nucleus and acts as a transcription regulator of androgen responsive genes by binding to the DNA [2,5]. Because of their anabolic effect, they are being investigated for use in cachexia, muscular dystrophies, Alzheimer’s disease, sarcopenia, and osteoporosis. Unlike traditional androgenic anabolic steroids, SARMs do not have androgenic side effects such as impaired fertility, acne, virilization, and prostate enlargement [2,4,7]. In fact, they have been investigated in the management of prostate cancer and benign prostate hyperplasia due to their antagonist properties [2]. Despite having many therapeutic uses, these agents are not regulated by FDA and are involved in several cases of hepatotoxicity [7]. The aforementioned presentation highlights an important and uncommon case of DILI in the setting of SARMs.

The exact mechanisms of hepatic injury caused by SARMs are yet to be deciphered. The DILIs can be dose-dependent (intrinsic) or dose-independent (idiosyncratic). Most DILI cases are secondary to idiosyncratic reactions, and drugs causing these reactions have a dose-dependent component [5]. Also, the rarity of reported cases relative to the degree of misuse and lack of association between the severity of the liver injury and the length of use points toward an idiosyncratic immune response. This information hints that the patient in the case discussed above had a liver injury either due to intrinsic cause or idiosyncratic reaction with a dose-dependent component. The mixed pattern of injury and histologic findings in all cases suggest damage to cholangiocytes and hepatocytes by the immune response [1]. In our patient, we used U.S. Drug-Induced Liver Injury Network (DILIN) causality and DILIN severity score to determine the likelihood and intensity of liver toxicity by drugs (SARMs). The scores were 2 and 3+, respectively, suggesting a moderate to severe DILI due to SARMs [3,8,9].

The DILI can have various histologic manifestations. According to various studies and DILIN, there are 18 discrete histologic patterns for DILI. They are categorized as acute cholestatic, chronic cholestatic, acute hepatitic, chronic hepatitic, granulomatous, microvesicular steatotic, cholestatic-hepatitic, macrovesicular steatotic, steatohepatitic, vascular injury, zonal necrosis, nonzonal necrosis, hepatocellular alteration, mixed or unclassified injury, nodular regenerative hyperplasia, minimal nonspecific changes, massive necrosis, and absolutely normal [10,11]. As suggested by various case reports, SARM-induced liver injury mostly manifest as a cholestatic pattern, as seen in our patient whose liver biopsy showed centrilobular biliary stasis [1,3-5,7,12].

Although SARMs’ therapeutic use is encouraging due to their tissue selectivity, this and other cases of their liver injury bring this tissue selectivity into question. As previously mentioned, SARMs can influence gene transcription by activating intracellular AR leading to androgen stimulated cell growth. As a result, they can have similar outcomes as AASs, resulting in three types of liver injury due to augmented cell growth, which include hepatic tumors, peliosis hepatis, and nodular regenerative hyperplasia. Due to the increase in the use of SARMs, it is essential to report these cases as early as possible to help recognize the offending agent and to understand its long-term effects [6].

Despite FDA’s warnings regarding safety concerns with SARMs, they are still being used and advertised as a bodybuilding supplement. They can be easily purchased online or over the counter as a prescription is not required. Apart from hepatotoxicity caused directly by SARMs, another concern with the use of these supplements is the unapproved drugs that are sometimes sold with them. According to a research conducted on 44 dietary supplements, 39% of them contained unapproved drugs other than SARMs. Further analysis revealed that only 52% of these supplements sold in the market had SARM, and remainder of the supplements were mislabeled. It is therefore highly possible that our patient may have used another unapproved and unlabeled substance while consuming SARM supplement [4].

This case among others raises questions and concerns about the safety of SARMs. Therefore, until more data are available, FDA and other concerned authorities should strictly regulate these products to prevent any further unwanted events [5,12].

## Conclusions

This case highlights an important issue of SARMs, which are small molecule, non-steroidal drugs that lead to tissue selective activities by binding as a ligand to ARs. They have anabolic effect on muscles and bones, but unlike traditional anabolic substances, SARMs do not have androgenic effects, and this property makes them ideal supplement among athletes and bodybuilders.

With numerous therapeutic benefits, these supplements come at a price. Multiple cases have been reported for liver injury secondary to these supplements. Therefore, it is high time that FDA and other concerned authorities should strictly regulate the manufacturing and sale of these drugs to avoid any undesirable side effects.
